# Labor Management and Neonatal Outcomes in Term Isolated Oligohydramnios Defined by the Single Deepest Vertical Pocket: A Retrospective Cohort Study

**DOI:** 10.3390/jcm15197338

**Published:** 2026-09-22

**Authors:** Emel Özalp, Kubilay Çanga, Özgür Volkan Akbulut, Türkan Dikici Aktaş, Şevki Çelen

**Affiliations:** 1 Department of Obstetrics and Gynecology, Ankara Etlik City Hospital, Ankara 06170, Türkiye; trkndkc1@gmail.com; 2 Department of Perinatology, Ankara Etlik City Hospital, Ankara 06170, Türkiye; kblycng@gmail.com (K.Ç.); akbulutvolkan@yahoo.com (Ö.V.A.); sevkicelen@yahoo.com (Ş.Ç.)

**Keywords:** oligohydramnios, amniotic fluid, pregnancy outcome

## Abstract

**Background/Objectives:** The clinical significance of isolated oligohydramnios at term may depend on the measurement method used. We assessed whether isolated oligohydramnios at term, defined by a single deepest vertical pocket (SDVP) below 2 cm, is associated with labor management, primary cesarean delivery, and short-term neonatal outcomes. **Methods:** This retrospective cohort study included 328 singleton pregnancies delivered at 37 + 0 to 41 + 6 weeks at a tertiary center between January 2023 and May 2026. All 128 exposed pregnancies met SDVP < 2 cm; 72 AFI-only cases with SDVP ≥ 2 cm were excluded from the primary analysis. The control group comprised 200 low-risk term pregnancies with SDVP ≥ 2 cm. Primary cesarean outcomes were evaluated among women without a previous cesarean delivery. Multivariable logistic regression estimated adjusted odds ratios (aORs) with 95% confidence intervals (CIs). **Results:** Labor induction was more frequent with SDVP-defined isolated oligohydramnios than with normal fluid (75.0% vs. 29.0%; *p* < 0.001). Among induced women, admission Bishop score, labor duration, and failed-induction rate were similar (6.3% vs. 5.2%; *p* = 1.000). Overall cesarean delivery, including repeat procedures, occurred in 30.5% versus 19.0% (*p* = 0.017). Primary cesarean delivery occurred in 25.8% and 18.5% (aOR, 1.67; 95% CI, 0.94–2.99), whereas primary cesarean delivery for fetal distress was more frequent (14.2% vs. 5.6%; aOR, 2.50; 95% CI, 1.05–5.94). Composite neonatal morbidity did not differ significantly (12.5% vs. 9.0%; aOR, 1.31; 95% CI, 0.61–2.78), nor did neonatal intensive care unit admission, cord pH, or neonatal death. **Conclusions:** At term, SDVP-defined isolated oligohydramnios was associated with more labor induction, a higher crude overall cesarean rate, and more primary cesarean delivery for fetal distress. No statistically significant adjusted association was detected for primary cesarean delivery or composite neonatal morbidity; the confidence intervals do not establish equivalence or exclude clinically important differences.

## 1. Introduction

Amniotic fluid supports fetal movement, lung development, temperature stability, and protection from umbilical cord compression. Near term, its volume reflects a dynamic balance among fetal urine production, swallowing, and intramembranous transfer, and low volume may signal placental dysfunction or fetal compromise [1]. Ultrasound assessment is therefore embedded in routine obstetric surveillance, particularly when fetal growth, placental function, or post-date pregnancy is a concern [2]. The four-quadrant amniotic fluid index (AFI) provided a reproducible semiquantitative framework, but threshold-based use also created a broad diagnostic category at the lower end of normal physiology [3].

Oligohydramnios is commonly identified by an AFI of 5 cm or less or a single deepest vertical pocket (SDVP) below 2 cm, but these methods do not identify identical populations. A meta-analysis of randomized trials found that AFI-based assessment increased the diagnosis of oligohydramnios, labor induction, and cesarean delivery for fetal distress without improving neonatal outcomes [4]. The multicenter SAFE trial similarly demonstrated more diagnoses and inductions with AFI than with SDVP, while neonatal intensive care unit (NICU) admission was unchanged [5]. Contemporary guidance therefore emphasizes interpretation of fluid measurements within the broader maternal–fetal clinical picture [6]. To reduce method-related overdiagnosis, the primary exposure in the present study was restricted to SDVP < 2 cm.

Management becomes especially difficult when low fluid is the only abnormal finding at term. Delivery is often favored because the fetal risk of continuing pregnancy is uncertain and the neonatal cost of term delivery is usually low. However, early-term birth remains associated with more neonatal respiratory and transitional morbidity than birth at 39 weeks, so the timing decision is not neutral [7]. A systematic review of term isolated oligohydramnios reported more intervention and cesarean delivery, but its only randomized comparison did not demonstrate a neonatal advantage of induction over expectant management [8]. Another meta-analysis found more obstetric intervention without clear differences in objective neonatal outcomes such as cord pH or NICU admission [9].

The observational literature remains heterogeneous because studies use different fluid criteria, gestational-age windows, exclusion rules, and thresholds for intervention. A systematic review separating complicated from uncomplicated pregnancies found higher rates of emergency cesarean delivery and selected neonatal outcomes in low-risk isolated cases, although effect estimates varied across studies [10]. A recent cohort defined by the single deepest pocket method reported more abnormal cardiotocography and cesarean delivery, particularly among nulliparous women, but no meaningful difference in perinatal morbidity [11]. In a large multicenter Chinese cohort, isolated oligohydramnios was associated with lower birth weight and more cesarean delivery, whereas perinatal death remained rare [12].

More recent evidence also suggests that risk may depend on the severity of fluid reduction and the fetal growth context. Severe isolated oligohydramnios has been linked to higher composite adverse outcomes than mild disease, although residual confounding by management cannot be excluded [13]. A 2025 meta-analysis reported an association with small-for-gestational-age birth, raising the possibility that some apparently isolated cases include subtle placental or growth abnormalities [14]. Placental pathology studies support this biological possibility but do not establish that delivery based on fluid measurement alone improves outcomes [15]. SDVP-based assessment and its association with perinatal outcomes have already been studied [5,11]. The incremental focus of the present study is to distinguish the frequency of induction from outcomes after induction has begun, while evaluating primary cesarean delivery in women without a previous cesarean delivery. We therefore evaluated labor management, primary cesarean delivery, and neonatal outcomes in a term cohort defined exclusively by SDVP < 2 cm, and separately examined induction-specific outcomes among induced women. The study addresses associations with clinical management and short-term neonatal outcomes; it does not validate a new fluid-measurement method or an individual risk-prediction model.

## 2. Materials and Methods

### 2.1. Study Design and Setting

We conducted a retrospective cohort study using hospital electronic records, including antenatal outpatient assessments, delivery-suite records, and neonatal records from a tertiary referral center. The source period was 1 January 2023 through 31 May 2026. Term pregnancy was defined as 37 + 0 through 41 + 6 weeks, consistent with contemporary term categories [16]. The study was prepared in accordance with the Strengthening the Reporting of Observational Studies in Epidemiology (STROBE) recommendations [17]. The unit of analysis was the pregnancy and its resulting neonate; only one delivery per eligible pregnancy was included.

Ankara Etlik City Hospital Scientific Research Evaluation and Ethics Committee approved the study protocol (protocol code AEŞH-BADEK2-2026-532 and date of approval: 30 June 2026). The approved protocol concerned retrospective analysis of routinely collected de-identified clinical data. No patient contact or study-specific examination, treatment, or intervention was planned, and the requirement for individual written informed consent was waived accordingly. The study was conducted in accordance with the Declaration of Helsinki.

### 2.2. Participants

Inclusion criteria were maternal age ≥18 years, a singleton cephalic pregnancy, and delivery at 37 + 0–41 + 6 weeks. For the primary exposed cohort, isolated oligohydramnios required SDVP < 2 cm at the index assessment, with intact membranes and no separate maternal or fetal indication independently requiring delivery.

Exclusion criteria were ruptured membranes; antenatally diagnosed fetal growth restriction; a hypertensive disorder; pregestational or gestational diabetes; a major fetal anomaly; a placental disorder; another medical or obstetric indication independently requiring delivery; non-cephalic presentation; or a mandatory indication for cesarean delivery.

The source exposed dataset contained 200 pregnancies meeting AFI ≤ 5 cm and/or SDVP < 2 cm. The primary exposure criterion was SDVP < 2 cm irrespective of the accompanying AFI value, and all 128 pregnancies retained in the primary exposed cohort met this SDVP criterion. The other 72 pregnancies met the AFI criterion only (AFI ≤ 5 cm with SDVP ≥ 2 cm); they were excluded from the primary analysis and were not reassigned to the control group. The comparison cohort comprised 200 eligible term, low-risk pregnancies with SDVP ≥ 2 cm selected by random sampling from the delivery registry of the same study period; no statistical matching or matched analysis was performed. Thus, the primary analytic sample comprised 328 pregnancies (128 exposed and 200 controls). The broader definition, comprising all 200 source exposed pregnancies and the same 200 controls, was used only in the separately identified sensitivity analysis. Sample size was determined by the available dataset rather than an a priori power calculation. Umbilical artery pH and base excess were available for 269 births (105 exposed and 164 controls), with similar availability proportions between groups. Biophysical profile scores were missing for 150 pregnancies and were not used as outcomes or adjustment variables. No statistical imputation was performed.

Maternal demographic and obstetric-history variables were linked to the index ultrasound and delivery record through the coded patient identifier. Gestational age was recorded in completed days and converted to weeks for presentation. Labor interventions and cesarean indications were obtained from delivery records, while neonatal outcomes were obtained from newborn and NICU records when applicable. Logic checks compared recorded delivery mode with cesarean and operative-vaginal indicators and compared NICU admission with length-of-stay entries. Analyses used only coded study records.

### 2.3. Exposure and Clinical Management

Ultrasound examinations were performed using a Voluson E8 system (GE Healthcare) by perinatology specialists and physicians undertaking perinatology subspecialty fellowship training. Exposure classification used the SDVP recorded at the index ultrasound examination. SDVP was the vertical depth, in centimeters, of the single largest amniotic-fluid pocket free of fetal parts and visible umbilical cord; SDVP < 2 cm defined the primary exposed group. Decisions regarding admission, surveillance, induction, cervical ripening, oxytocin, amniotomy, analgesia, and cesarean delivery were made by the responsible clinical team under routine institutional practice. In the recorded cohort, women with a Bishop score ≤ 5 received cervical ripening with either a balloon catheter or dinoprostone, whereas those with a more favorable cervix proceeded without a ripening agent; subsequent amniotomy and oxytocin were used according to clinical circumstances.

### 2.4. Outcomes

Maternal and labor outcomes included induction of labor, Bishop score and cervical dilation at the start of induction, cervical-ripening method, oxytocin use, amniotomy, epidural analgesia, labor duration, failed induction, mode of delivery, non-reassuring cardiotocography, meconium-stained fluid, postpartum hemorrhage, and recorded maternal complications. Overall cesarean delivery included both primary and repeat procedures. Primary cesarean delivery was defined as cesarean delivery among women without a previous cesarean delivery; primary cesarean delivery for fetal distress was evaluated in the same no-previous-cesarean population. Failed induction was defined as failure to enter active labor within 48 h after initiation of induction and was evaluated only among women who underwent induction. Women with a previous cesarean delivery contributed to the overall delivery and neonatal analyses but were excluded from all primary-cesarean analyses.

Neonatal outcomes included birth weight, recorded birth-weight percentile, small-for-gestational-age birth (birth weight below the recorded 10th percentile), sex, 1 and 5 min Apgar scores, umbilical artery pH and base excess when available, NICU admission and length of stay, transient tachypnea of the newborn, respiratory distress syndrome, respiratory support, surfactant treatment, meconium aspiration syndrome, suspected sepsis or antibiotic treatment, and neonatal death.

The composite neonatal morbidity outcome was a prespecified, study-specific binary endpoint, not a validated clinical score or a guideline-defined syndrome. It was positive when at least one of the following was recorded: 5 min Apgar score < 7; umbilical artery pH < 7.10 when sampled; NICU admission; transient tachypnea of the newborn or respiratory distress syndrome; need for respiratory support; or meconium aspiration syndrome. Each neonate contributed at most one composite event, irrespective of the number of components present. The definition combines immediate neonatal condition, cord-gas findings, respiratory morbidity, and escalation of care, and the individual components are reported separately in Table 1. A low Apgar score was not interpreted as a stand-alone diagnosis of asphyxia, consistent with AAP/ACOG guidance [18].

NICU admission was a separate, all-cause component and was not restricted to the respiratory diagnoses listed above. At Ankara Etlik City Hospital, neonatal admission decisions followed institutional NICU admission protocols, with clinical signs of respiratory distress and/or a low pH among the indications considered. These indications were not an exhaustive list of reasons for admission or transfer. An admission for any other indication also contributed to the composite through the NICU component, without assigning an undocumented diagnosis. Detailed admission and transfer thresholds and the primary indication for each NICU admission could not be retrieved from the retrospective records. The study threshold of umbilical artery pH < 7.10 defines the biochemical outcome component and should not be interpreted as a verified institutional admission cutoff. AAP standards describe neonatal care capabilities and institution-specific triage, stabilization, and transfer policies; they do not define or validate this study-specific composite [19]. NICU admission is therefore interpreted as a care-utilization outcome influenced by local practice rather than a uniform measure of severe neonatal illness.

Cord pH was available for 269 of 328 neonates (105 exposed and 164 controls) and was unavailable for 59 (23 exposed and 36 controls). The biochemical component was evaluated only when a pH result was available; no missing pH value was imputed or treated as a measured normal result. A neonate without an available pH could still meet the composite through any documented clinical component, including all-cause NICU admission. Because low pH and respiratory distress were among the clinical indications for admission, the NICU component may capture some affected infants without a recorded cord-gas result. However, NICU admission was not used to infer acidemia and cannot replace a missing pH measurement. When neither a pH result nor another positive component was recorded, the endpoint represented no recorded composite event, not confirmed absence of morbidity or a normal acid–base status. The composite therefore describes recorded morbidity with incomplete biochemical ascertainment; pH-specific results use the available-case denominators of 105 and 164.

### 2.5. Statistical Analysis

Continuous variables were summarized as mean with standard deviation or median with interquartile range according to distribution and clinical scale. Categorical variables were summarized as count and percentage. In the tables, continuous data are presented as mean ± standard deviation or median [interquartile range], and categorical data as n (%) or n/N (%) when an outcome-specific denominator is needed. Group comparisons used Welch’s t test for approximately normally distributed continuous variables, the Mann–Whitney U test for skewed or ordinal variables, and the Pearson chi-square or Fisher exact test for categorical variables, as appropriate. Overall *p* values for mode of delivery and cervical-ripening method compare all listed categories. For mode of delivery, Pearson chi-square tests compared the three categories jointly and, separately, all cesarean deliveries versus non-cesarean deliveries; no continuity correction was applied. SDVP is the exposure-defining measurement and is included in Table 1 to describe the groups, rather than to establish baseline comparability. All tests were two-sided, and *p* < 0.05 was considered statistically significant.

Logistic regression estimated odds ratios (ORs) and 95% confidence intervals (CIs) for induction of labor, primary cesarean delivery, primary cesarean delivery for fetal distress, composite neonatal morbidity, and NICU admission. For adjusted outcomes, candidate covariates were first examined in univariable logistic regression. Maternal age, body mass index, nulliparity, in vitro fertilization, smoking, gestational age at delivery, estimated fetal weight at the index examination, and birth weight were considered; variables with *p* < 0.20 entered the outcome-specific multivariable model, while SDVP-defined isolated oligohydramnios was forced into each model. Estimated fetal weight and birth weight were not entered together. Previous cesarean delivery was not used as a covariate because primary cesarean outcomes were restricted to women without a previous cesarean delivery.

The final adjusted models included maternal age, nulliparity, and smoking for primary cesarean delivery; maternal age and birth weight for primary cesarean delivery for fetal distress; body mass index and gestational age at delivery for composite neonatal morbidity; and body mass index and birth weight for NICU admission. Induction was considered a potential management-pathway variable rather than a baseline confounder and was added only in sensitivity models for the two primary cesarean outcomes. A further, separately reported sensitivity analysis used the broader source definition of AFI ≤ 5 cm and/or SDVP < 2 cm (200 exposed pregnancies, including the 72 AFI-only cases, and the same 200 controls). The primary estimates in Table 1, Table 2, Table 3 and Table 4 use the SDVP-defined cohort, not this broader cohort. An adjusted failed-induction model was not fitted because only nine events occurred in the primary SDVP-defined cohort. Analyses were performed with Python version 3.13 (Python Software Foundation, Wilmington, DE, USA) using the SciPy 1.17.0 and statsmodels 0.14.6 packages. Regression convergence was verified, continuous covariates were retained on their continuous scale, and secondary outcomes were interpreted as exploratory.

The study was designed to evaluate associations between SDVP-defined isolated oligohydramnios, labor management, and recorded short-term neonatal outcomes. Logistic regression was used for covariate-adjusted association estimates, not to develop an individual risk-prediction tool. ROC/AUC, calibration, and decision-curve analyses address the performance and clinical utility of prediction models and were outside this study objective. A separate prediction study would require a defined clinical decision point, predictors available at that time, an appropriate sampling frame, adequate sample-size planning, and validation of discrimination, calibration, and clinical utility [20,21]. Forest plots display the ORs and 95% CIs from the main and induction-adjusted sensitivity models.

## 3. Results

### 3.1. Cohort Characteristics

The primary analysis included 328 pregnancies: 128 with documented SDVP < 2 cm and 200 controls with SDVP ≥ 2 cm. The 72 AFI-only pregnancies were not included in this cohort or in the primary descriptive tables. Maternal age, body mass index, gravida, parity, nulliparity, conception method, smoking, and previous cesarean delivery were similar between groups. The exposed group underwent the index assessment 3.52 days earlier and delivered 4.47 days earlier on average. Early-term delivery occurred in 54.7% versus 22.0% (*p* < 0.001). Median SDVP was 1.30 versus 4.55 cm (*p* < 0.001), and estimated fetal weight at the index examination was 204 g lower in the exposed group (Table 2).

**Table 2:** Maternal and pregnancy characteristics.

Characteristic	SDVP-Defined Isolated Oligohydramnios (SDVP < 2 cm; *n* = 128)	Control (SDVP ≥ 2 cm; *n* = 200)	*p* Value
Maternal age, years	28.75 ± 4.54	28.31 ± 4.19	0.373
Body mass index, kg/m^2^	27.12 ± 4.28	27.41 ± 4.10	0.556
Gravida	2 [1–3]	2 [1–3]	0.243
Parity	1 [0–1]	1 [0–1]	0.717
Nulliparity	59 (46.1)	96 (48.0)	0.736
In vitro fertilization	5 (3.9)	9 (4.5)	0.795
Smoking	12 (9.4)	20 (10.0)	0.852
Previous cesarean delivery	8 (6.3)	22 (11.0)	0.145
Gestational age at index scan, weeks	38.66 ± 0.95	39.16 ± 0.93	<0.001
Gestational age at delivery, weeks	38.82 ± 0.96	39.46 ± 0.94	<0.001
Early-term delivery (37 + 0–38 + 6 weeks)	70 (54.7)	44 (22.0)	<0.001
Single deepest vertical pocket, cm	1.30 [1.10–1.50]	4.55 [3.80–5.23]	<0.001
Estimated fetal weight at index scan, g	3098 ± 332	3302 ± 305	<0.001

Primary SDVP-defined cohort only. All 128 exposed pregnancies had SDVP < 2 cm; the 72 AFI-only pregnancies were excluded. Data presentation and statistical tests are described in Section 2.5. SDVP: Single deepest vertical pocket.

### 3.2. Labor and Delivery Outcomes

Induction of labor was performed in 96 of 128 women (75.0%) with SDVP-defined isolated oligohydramnios and 58 of 200 controls (29.0%; *p* < 0.001), corresponding to an unadjusted OR of 7.34 (95% CI, 4.44–12.15). Oxytocin use was more frequent in the exposed cohort (66.4% vs. 50.5%; *p* = 0.005), while the difference in amniotomy did not reach statistical significance (63.3% vs. 53.0%; *p* = 0.067); epidural use was similar. Among induced women, the median admission Bishop score was 4 in both groups. Cervical-ripening methods were similarly distributed (overall *p* = 0.781), labor duration was 8.85 versus 9.05 h (*p* = 0.554), and failed induction occurred in six of 96 exposed women (6.3%) and three of 58 controls (5.2%; *p* = 1.000).

The overall distribution of spontaneous vaginal, operative vaginal, and cesarean delivery did not reach statistical significance in the three-category comparison (*p* = 0.055). In the separate binary comparison, overall cesarean delivery, including repeat procedures, was more frequent in the exposed group: 39 of 128 women (30.5%) versus 38 of 200 controls (19.0%; *p* = 0.017). The 39 exposed-group cesareans comprised 31 primary and eight repeat procedures. Spontaneous vaginal delivery occurred in 85 of 128 exposed women (66.4%), and operative-vaginal delivery in four (3.1%). Among women without a previous cesarean delivery, primary cesarean delivery occurred in 31 of 120 exposed women (25.8%) and 33 of 178 controls (18.5%; *p* = 0.133). Primary cesarean delivery for fetal distress was more frequent with isolated oligohydramnios (14.2% vs. 5.6%; *p* = 0.012), as were non-reassuring cardiotocography (18.0% vs. 10.0%; *p* = 0.037) and meconium-stained fluid (18.0% vs. 9.0%; *p* = 0.017). Postpartum hemorrhage and recorded maternal complications were uncommon and similar (Table 3).

### 3.3. Neonatal Outcomes

Mean birth weight was 3135 ± 293 g in the isolated oligohydramnios group and 3344 ± 279 g in controls, a mean difference of −209 g (*p* < 0.001). The median recorded birth-weight percentile was lower (48.65 vs. 58.20; *p* = 0.005), but small-for-gestational-age birth did not differ significantly (4.7% vs. 2.0%; *p* = 0.197). Median Apgar scores were high in both groups. A 5 min Apgar score < 7 occurred in 1.6% versus 0.5% (*p* = 0.563). Among neonates with cord-gas results, mean umbilical artery pH was 7.266 versus 7.276 (*p* = 0.069), and pH < 7.10 occurred in three and one neonates, respectively (*p* = 0.303). Mean base excess was also similar (−3.52 vs. −3.04 mmol/L; *p* = 0.062).

NICU admission occurred in 7.0% of exposed neonates and 5.5% of controls (*p* = 0.572). Transient tachypnea occurred in 4.7% versus 3.0%, and respiratory support was required in 6.3% versus 5.5%; neither difference was significant. Respiratory distress syndrome, surfactant treatment, meconium aspiration syndrome, and suspected sepsis or antibiotic treatment were uncommon and similar. There were no neonatal deaths. Composite neonatal morbidity occurred in 16 exposed neonates (12.5%) and 18 controls (9.0%; *p* = 0.310) (Table 3).

**Table 3:** Neonatal outcomes.

Outcome	SDVP-Defined Isolated Oligohydramnios (SDVP < 2 cm; *n* = 128)	Control (SDVP ≥ 2 cm; *n* = 200)	*p* Value
Birth weight, g	3135 ± 293	3344 ± 279	<0.001
Recorded birth-weight percentile	48.65 [28.15–65.70]	58.20 [38.58–75.58]	0.005
Small for gestational age	6 (4.7)	4 (2.0)	0.197
Male sex	63 (49.2)	105 (52.5)	0.562
1 min Apgar score <7	4 (3.1)	5 (2.5)	0.740
5 min Apgar score <7	2 (1.6)	1 (0.5)	0.563
Umbilical artery pH	7.266 ± 0.048	7.276 ± 0.034	0.069
Umbilical artery pH <7.10	3/105 (2.9)	1/164 (0.6)	0.303
Base excess, mmol/L	−3.52 ± 2.24	−3.04 ± 1.69	0.062
NICU admission	9 (7.0)	11 (5.5)	0.572
NICU stay among admitted, days	2 [2–3]	3 [2–3]	0.302
Transient tachypnea of the newborn	6 (4.7)	6 (3.0)	0.549
Respiratory distress syndrome	0	1 (0.5)	1.000
Any respiratory support	8 (6.3)	11 (5.5)	0.777
Surfactant treatment	0	1 (0.5)	1.000
Meconium aspiration syndrome	0	1 (0.5)	1.000
Suspected sepsis/antibiotic treatment	3 (2.3)	3 (1.5)	0.682
Neonatal death	0	0	–
Composite neonatal morbidity outcome	16 (12.5)	18 (9.0)	0.310

Primary SDVP-defined cohort only. Umbilical artery pH and base excess were available for 105 exposed neonates and 164 controls; pH was unavailable for 23 and 36, respectively. The composite is positive for at least one recorded component: 5 min Apgar score < 7, umbilical artery pH < 7.10 when available, all-cause NICU admission, transient tachypnea or respiratory distress syndrome, respiratory support, or meconium aspiration syndrome. Each neonate is counted once. Infants without a pH result can meet the composite through another recorded component. No recorded event is not proof of normal pH; NICU admission neither imputes nor replaces pH. Data presentation and statistical tests are described in Section 2.5. NICU: Neonatal intensive care unit; SDVP: Single deepest vertical pocket.

### 3.4. Regression Analyses

No statistically significant adjusted association was detected between SDVP-defined isolated oligohydramnios and primary cesarean delivery (unadjusted OR, 1.53; 95% CI, 0.88–2.67; adjusted OR [aOR], 1.67; 95% CI, 0.94–2.99; *p* = 0.083). The association with primary cesarean delivery for fetal distress remained significant after adjustment (unadjusted OR, 2.77; 95% CI, 1.22–6.29; aOR, 2.50; 95% CI, 1.05–5.94; *p* = 0.039). No statistically significant association was detected with composite neonatal morbidity (aOR, 1.31; 95% CI, 0.61–2.78; *p* = 0.487) or NICU admission (aOR, 1.11; 95% CI, 0.42–2.93; *p* = 0.833). After induction was added to sensitivity models, estimates attenuated for primary cesarean delivery (aOR, 1.09; 95% CI, 0.57–2.11; *p* = 0.792) and primary cesarean delivery for fetal distress (aOR, 1.75; 95% CI, 0.68–4.50; *p* = 0.245). In the broader AFI ≤ 5 cm and/or SDVP < 2 cm sensitivity cohort, the adjusted associations were 1.81 (95% CI, 1.09–3.03; *p* = 0.023) for primary cesarean delivery and 2.56 (95% CI, 1.16–5.64; *p* = 0.020) for primary cesarean delivery for fetal distress, while neonatal outcome models remained non-significant (Table 4).

**Table 4:** Associations between SDVP-defined isolated oligohydramnios and selected outcomes.

Outcome	Unadjusted OR (95% CI)	Adjusted OR (95% CI)	Adjusted *p* Value
Induction of labor	7.34 (4.44–12.15)	Not modeled	–
Primary cesarean delivery	1.53 (0.88–2.67)	1.67 (0.94–2.99) ^1^	0.083
Primary cesarean for fetal distress	2.77 (1.22–6.29)	2.50 (1.05–5.94) ^2^	0.039
Composite neonatal morbidity outcome	1.44 (0.71–2.95)	1.31 (0.61–2.78) ^3^	0.487
NICU admission	1.30 (0.52–3.23)	1.11 (0.42–2.93) ^4^	0.833

Primary SDVP-defined cohort only; the control group is the reference. Primary-cesarean models include 120 exposed women and 178 controls without previous cesarean delivery. Neonatal models use the 128 exposed pregnancies and 200 controls. ^1^ Adjusted for maternal age, nulliparity, and smoking. ^2^ Adjusted for maternal age and birth weight. ^3^ Adjusted for body mass index and gestational age at delivery. ^4^ Adjusted for body mass index and birth weight. Candidate covariates with univariable *p* < 0.20 entered outcome-specific models; isolated oligohydramnios was forced into each model. Sensitivity models additionally including induction yielded adjusted ORs of 1.09 (95% CI, 0.57–2.11; *p* = 0.792) for primary cesarean delivery and 1.75 (95% CI, 0.68–4.50; *p* = 0.245) for primary cesarean delivery for fetal distress. CI: Confidence interval; NICU: Neonatal intensive care unit; OR: Odds ratio; SDVP: Single deepest vertical pocket.

Figure 1 displays the unadjusted and adjusted associations in the primary SDVP-defined cohort. Figure 2 displays the cesarean estimates before and after adding induction to the adjusted models.

## 4. Discussion

In this term cohort defined by SDVP < 2 cm, isolated oligohydramnios was associated with a marked increase in labor induction, more non-reassuring cardiotocography, and more primary cesarean delivery for fetal distress. Overall cesarean delivery, including repeat procedures, was more frequent in the crude comparison (30.5% vs. 19.0%; *p* = 0.017). This all-participant result is distinct from the primary-cesarean analysis restricted to women without a previous cesarean delivery, for which the adjusted association did not reach statistical significance. No statistically significant increase was detected in the composite neonatal morbidity outcome or the reported individual neonatal outcomes. These null findings do not demonstrate equivalence: the aOR for composite neonatal morbidity was 1.31, with a 95% CI of 0.61–2.78, and no neonatal deaths were observed. Among women who underwent induction, cervical favorability, labor duration, and failed-induction rates showed no statistically significant differences. The results describe an intervention burden without a statistically clear neonatal morbidity difference, while leaving substantial uncertainty about uncommon serious outcomes.

The pattern of greater intervention is consistent with prior observational evidence. Ashwal and colleagues found that term isolated oligohydramnios was associated with more induction and cesarean delivery for non-reassuring fetal heart-rate patterns [22]. By contrast, Naveiro-Fuentes et al. reported broadly reassuring perinatal outcomes and identified small-for-gestational-age birth as the principal difference [23]. In the RADIUS secondary analysis, Zhang et al. found no evidence that isolated low fluid impaired growth or increased adverse perinatal outcomes [24]. Divergence across studies may reflect the fluid-measurement method, thresholds for delivery, control selection, and the extent to which subtle growth or placental abnormalities were excluded.

Our adjusted results require careful interpretation. Restricting primary-cesarean analysis to women without a previous cesarean delivery separates this outcome from repeat procedures and from the influence of prior uterine surgery. Adding induction to the models attenuated both cesarean estimates. Because induction occurs after diagnosis and may lie on the management pathway, this sensitivity analysis should not be interpreted as an ordinary confounding control; rather, the attenuation is compatible with a contribution from management decisions after diagnosis, but does not quantify a mediated effect or establish causation. More frequent cardiotocographic concern could reflect true susceptibility to cord compression, lower tolerance of labor, or a lower threshold for operative delivery in a pregnancy already labeled at risk.

The absence of a clear neonatal signal is biologically plausible when oligohydramnios is genuinely isolated. Seol et al. found that hourly fetal urine production was not reduced in isolated term oligohydramnios, whereas it was lower when fetal growth restriction was present, supporting the concept that low fluid alone may not always indicate impaired fetal renal perfusion [25]. In our cohort, cord pH, clinically low Apgar scores, respiratory outcomes, and NICU use were similar, and exposed neonates weighed 209 g less but were also delivered approximately 4.5 days earlier. Although residual placental differences cannot be excluded, the absence of a significant increase in small-for-gestational-age birth argues against severe unrecognized growth restriction as the sole explanation.

The induced-only analysis adds clinically useful information. Tahmina et al. compared induction outcomes in isolated and complicated term oligohydramnios and reported broadly similar maternal and perinatal outcomes apart from birth weight [26]. Kansu-Celik et al. reported acceptable cervical ripening with dinoprostone in women with isolated oligohydramnios and an unfavorable cervix [27], while earlier work by Manzanares et al. questioned whether isolated oligohydramnios should automatically be treated as an indication for induction [28]. In our cohort, induction was much more frequent after diagnosis, yet induced groups began with similar Bishop scores and had similar labor duration, ripening requirements, and failed-induction rates. The small number of failed inductions does not support a precise estimate of a difference, and these findings should not be interpreted as evidence of equivalent induction success. The contribution is a description of induction processes in the selected cohort, not a validation of SDVP as a new diagnostic method.

Measurement strategy is central to clinical translation. AFI classifies more pregnancies as oligohydramnios than SDVP and can increase intervention without a demonstrated neonatal benefit. An earlier meta-analysis found only a weak association between low intrapartum AFI and neonatal acidemia, although low AFI was associated with cesarean delivery for fetal distress and a 5 min Apgar score < 7 [29]. We therefore used SDVP < 2 cm for the primary analysis and retained the original AFI-or-SDVP definition only as a sensitivity analysis; the stronger total primary-cesarean association in the broader cohort shows that the association is sensitive to the exposure definition. This was not a randomized comparison of AFI and SDVP and does not independently establish the superiority of either measurement strategy. Future studies should report the diagnostic method, actual fluid value, persistence on repeat assessment, placental and fetal Doppler findings, and severity category. A pragmatic randomized trial comparing protocolized surveillance with delivery, stratified by SDVP severity and gestational week, would be more informative.

The practical implication is not that term isolated oligohydramnios should be ignored. Rather, the diagnosis should prompt confirmation of measurement quality and structured assessment for membrane rupture, impaired growth, hypertensive disease, placental dysfunction, and fetal compromise. When these conditions are absent, counseling should separate the uncertain fetal benefit of delivery from the predictable increase in induction exposure, recognizing that the absolute differences in severe neonatal events were small and that the confidence intervals remain compatible with clinically important differences in either direction. If induction is chosen, our data suggest that low fluid alone need not imply a longer labor or a higher probability of failed induction. Shared decision-making should incorporate gestational week, SDVP severity, cervical status, fetal testing, prior obstetric history, and local monitoring capacity.

The strengths of this study include isolation criteria that excluded major maternal and fetal conditions, capture of labor processes together with objective neonatal measures, and evaluation of cesarean outcomes in women without a previous cesarean delivery. Failed induction was calculated only among women exposed to induction, and outcome-specific covariate screening, pathway sensitivity models for induction, and a broad-definition sensitivity analysis made the analytic assumptions explicit. Several limitations should also be acknowledged. The retrospective, single-center design permits selection bias, misclassification, and residual confounding, and controls were randomly sampled rather than matched. Failed induction was taken from chart coding and could not be independently reconstructed from time-stamped labor records. Clinical teams were aware of the fluid diagnosis, so surveillance and decision thresholds could not be blinded. Examinations were performed by both perinatology specialists and subspecialty fellows; the retrospective records did not support an assessment of interobserver measurement reproducibility. Restricting the primary analysis to SDVP < 2 cm excluded AFI-only cases and reduced statistical precision, and the study was not powered for rare outcomes such as severe acidemia, meconium aspiration syndrome, stillbirth, or neonatal death. Cord blood gases were unavailable for 59 of 328 births (18.0%), although availability was balanced between groups, and long-term infant follow-up was not available.

The composite combines clinical findings of different severity with NICU admission, an outcome influenced by local practice, and should not be equated with severe neonatal morbidity. Although respiratory distress and/or low pH were among the indications for NICU admission under institutional protocols, detailed admission/transfer thresholds and the primary reason for each admission were unavailable in the retrospective records. We could therefore not provide a complete diagnosis-specific admission breakdown or assess adherence to each protocol criterion. The respiratory and biochemical components overlap with some admission indications, and all-cause NICU admission may capture some affected infants lacking a recorded cord pH. The extent of this overlap could not be quantified, however, and it does not compensate for the missing biochemical measurements. Acidemia unaccompanied by another recorded component may have been missed; similar pH availability between groups does not exclude ascertainment bias.

Outcome-specific covariate selection and the limited numbers of events also increase model uncertainty. The study was planned to assess associations rather than to develop or validate a neonatal prediction model. There were 34 recorded composite events and 20 NICU admissions, and the selected exposed/control ratio does not establish absolute risk in an unselected obstetric population. Moreover, gestational age at delivery and birth weight would not be available at an earlier antenatal decision point. A future prediction study should define its intended clinical use, select predictors available at that time, plan an adequate sample size, and validate discrimination, calibration, and clinical utility [20,21].

## 5. Conclusions

In the primary cohort, which included only exposed pregnancies with SDVP <2 cm and excluded all 72 AFI-only cases, isolated oligohydramnios was associated with more labor induction, a higher crude overall cesarean rate including repeat procedures, and more primary cesarean delivery for fetal distress. No statistically significant adjusted association was detected for primary cesarean delivery or composite neonatal morbidity. The wide confidence intervals and low numbers of serious events preclude a conclusion of equivalence or proof that expectant management is safe. The induced-only findings describe comparable observed labor measures without establishing equivalent induction success, and attenuation after including induction is compatible with management-pathway effects without proving mediation. These are observational association findings, not a validated neonatal prediction model or a basis for replacing clinical surveillance and delivery guidance. Prospective studies with consistent SDVP criteria, standardized outcome ascertainment and management, and adequate assessment of uncommon neonatal outcomes are needed.

## Figures and Tables

**Figure 1:**
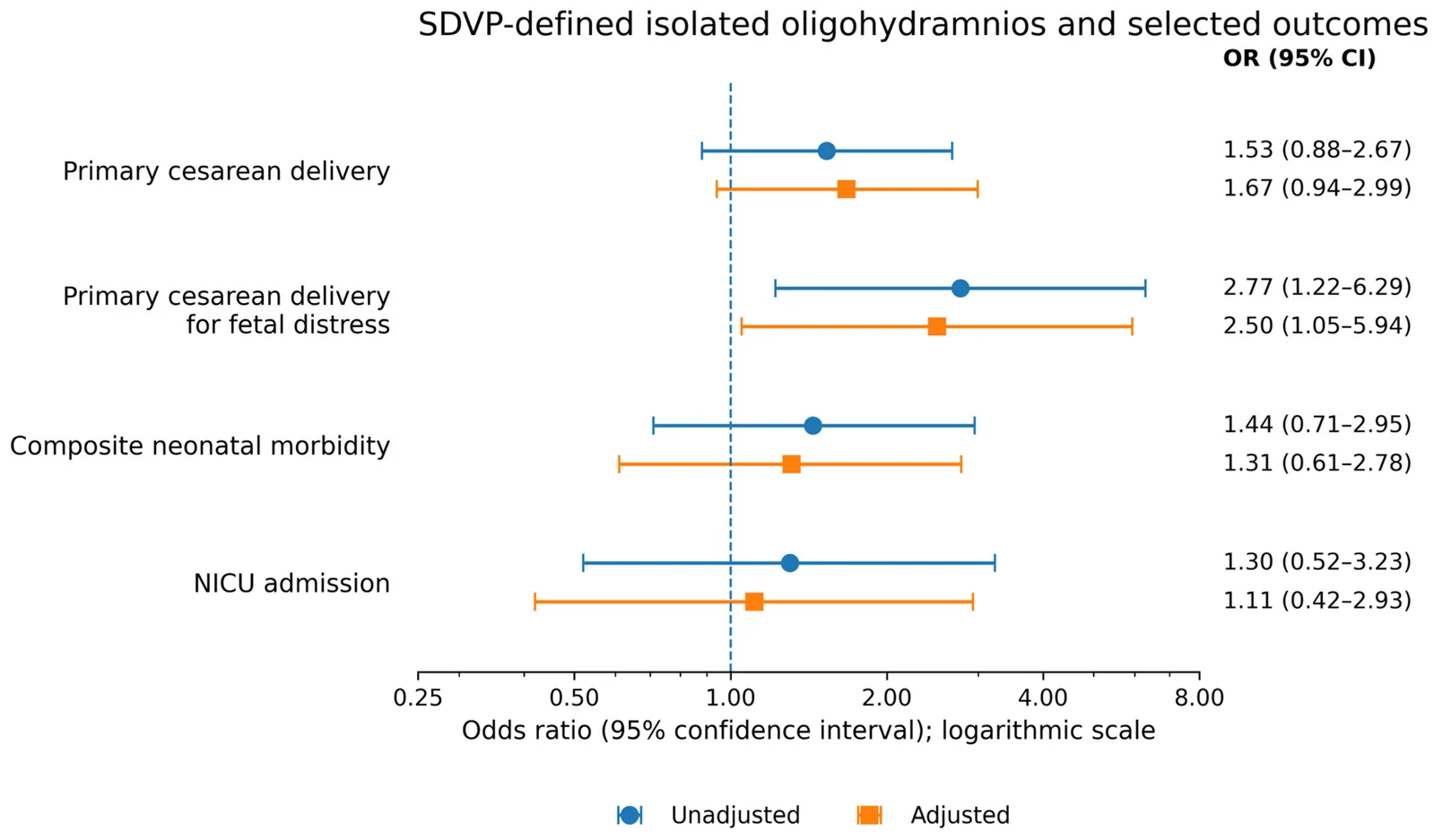
Unadjusted and adjusted associations between SDVP-defined isolated oligohydramnios and selected outcomes. Points indicate ORs and horizontal bars indicate 95% CIs on a logarithmic scale; the dashed line marks OR = 1. Primary cesarean outcomes are restricted to women without a previous cesarean delivery (120 exposed; 178 controls); neonatal outcomes use 128 exposed pregnancies and 200 controls. Adjustment sets are those specified in Table 4. Each row is a separate outcome, not a pooled meta-analysis. Estimates are reproduced from Table 4 and have not been re-estimated for this figure.

**Figure 2:**
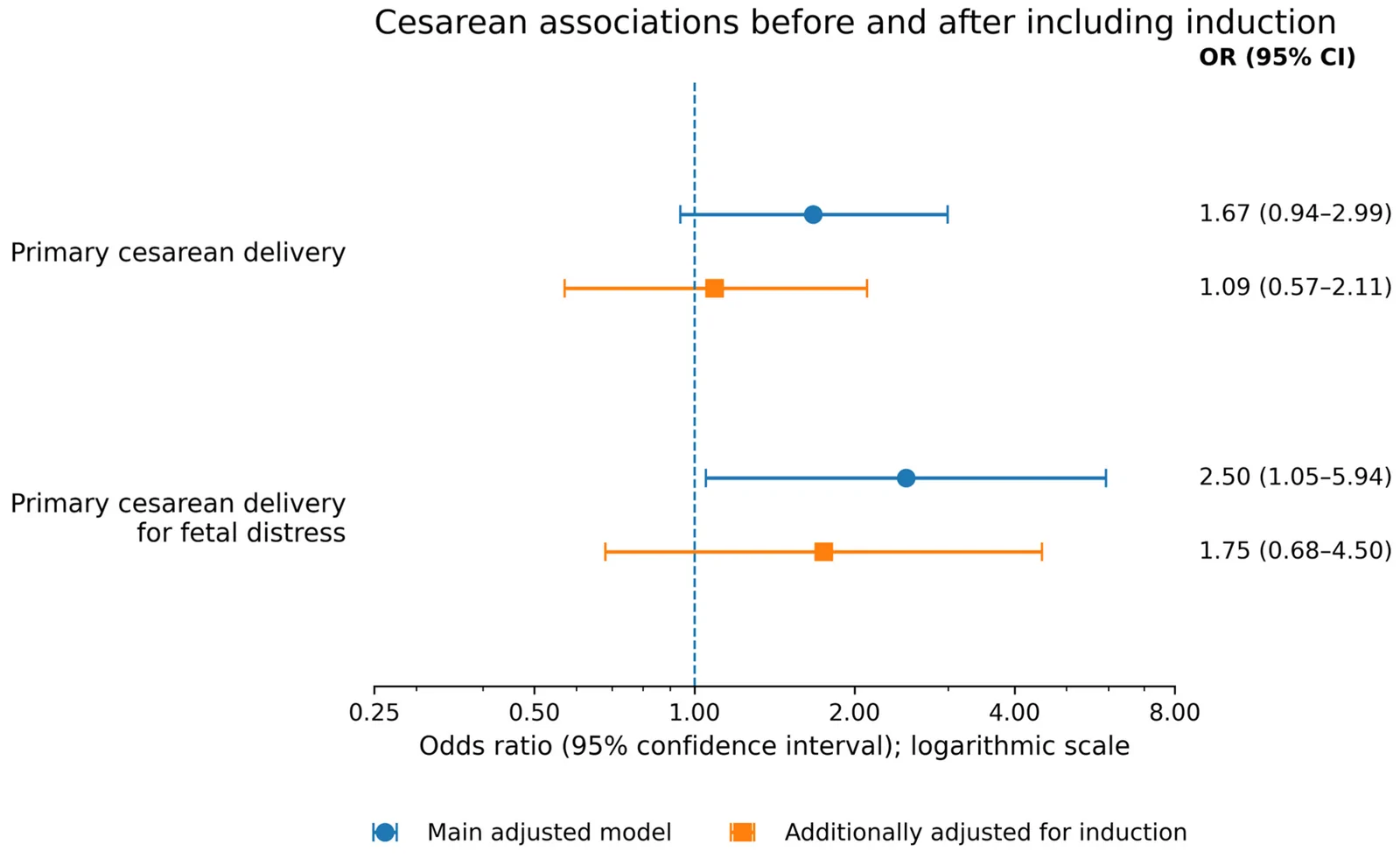
Induction-adjusted sensitivity analyses for primary cesarean outcomes in the SDVP-defined cohort. The main adjusted models are compared with the same models additionally including induction. All estimates use women without a previous cesarean delivery (120 exposed; 178 controls). The main primary-cesarean model adjusts for maternal age, nulliparity, and smoking; the fetal-distress model adjusts for maternal age and birth weight. Induction may lie on the post-diagnosis management pathway, so attenuation does not establish mediation or causation. Points and bars indicate aORs and 95% CIs; the dashed line marks aOR = 1. Estimates are reproduced from Section 3.4 and Table 4 footnote.

**Table 1:** Labor and maternal delivery outcomes.

Outcome	SDVP-Defined Isolated Oligohydramnios (SDVP < 2 cm; *n* = 128)	Control (SDVP ≥ 2 cm; *n* = 200)	*p* Value
**All participants**	***n*** **= 128**	***n*** **= 200**	
Induction of labor	96/128 (75.0)	58/200 (29.0)	<0.001
Oxytocin use	85/128 (66.4)	101/200 (50.5)	0.005
Amniotomy	81/128 (63.3)	106/200 (53.0)	0.067
Epidural analgesia	38/128 (29.7)	65/200 (32.5)	0.592
Mode of delivery			0.055
Spontaneous vaginal	85/128 (66.4)	156/200 (78.0)	
Operative vaginal	4/128 (3.1)	6/200 (3.0)	
Cesarean	39/128 (30.5)	38/200 (19.0)	0.017
Primary cesarean delivery	31/120 (25.8)	33/178 (18.5)	0.133
Primary cesarean for fetal distress	17/120 (14.2)	10/178 (5.6)	0.012
Non-reassuring cardiotocography	23/128 (18.0)	20/200 (10.0)	0.037
Meconium-stained fluid	23/128 (18.0)	18/200 (9.0)	0.017
Postpartum hemorrhage	5/128 (3.9)	5/200 (2.5)	0.520
Any recorded maternal complication	8/128 (6.3)	7/200 (3.5)	0.245
**Induced participants only**	***n*** **= 96**	***n*** **= 58**	
Bishop score at induction	4 [3–5]	4 [3–5]	0.209
Cervical dilation at induction, cm	0.75 [0.38–1.50]	1.00 [0.13–1.50]	0.317
Cervical-ripening method			0.781
Balloon	30 (31.3)	16 (27.6)	
Dinoprostone	53 (55.2)	32 (55.2)	
No ripening required	13 (13.5)	10 (17.2)	
Labor duration, hours	8.85 [6.90–11.63]	9.05 [6.70–10.58]	0.554
Failed induction	6 (6.3)	3 (5.2)	1.000

Primary SDVP-defined cohort only. Overall cesarean delivery includes primary and repeat procedures. The mode-of-delivery *p* value compares all three categories; the cesarean-row *p* value compares cesarean versus non-cesarean delivery. Primary cesarean outcomes include women without a previous cesarean delivery (120 exposed; 178 controls). Induction-specific outcomes include induced women only (96 exposed; 58 controls). Data presentation and statistical tests are described in Section 2.5. SDVP: Single deepest vertical pocket.

## Data Availability

The datasets generated and/or analyzed during the current study are not publicly available due to ethical and privacy considerations but are available from the corresponding author upon reasonable request, subject to appropriate ethical and institutional approval.

## Abbreviations

The following abbreviations are used in this manuscript:

AFI	Amniotic fluid index
aOR	Adjusted odds ratio
CI	Confidence interval
NICU	Neonatal intensive care unit
OR	Odds ratio
SDVP	Single deepest vertical pocket
STROBE	Strengthening the Reporting of Observational Studies in Epidemiology
